# Amphibian Chytrid Fungus Infection Influences Calling Investment in Male Brown Toadlets

**DOI:** 10.1002/ece3.72354

**Published:** 2025-10-20

**Authors:** Ewan S. Auld, Aimee J. Silla, Phillip G. Byrne

**Affiliations:** ^1^ School of Science, Environmental Futures University of Wollongong Wollongong New South Wales Australia

**Keywords:** amphibian, chytrid fungus, disease, frog calling, sexual advertisement, terminal investment

## Abstract

The sublethal effects of infection and disease on male advertisement behaviour remain poorly understood. The ‘terminal investment hypothesis’ proposes that infected organisms will increase reproductive investment when their future reproductive prospects decline. Evidence to support this hypothesis has come from reports that some anuran amphibians respond to infection with the amphibian chytrid fungus pathogen by elevating calling activity. However, the generality of this response remains unclear, justifying studies in more anuran species. The aim of the present study was to provide a preliminary examination of the terminal investment hypothesis by investigating the relationship between chytrid fungus infection status and male calling behaviour in wild Australian brown toadlets ( *Pseudophryne bibronii* ). Across four populations, we examined correlations between infection status and spectral and temporal properties of the biphasic advertisement call, while also accounting for interrelationships with intrinsic and extrinsic factors known to influence calling behaviour. We found that infected males produced calls with more pulses compared to uninfected males. The association we report supports the terminal investment hypothesis and has the potential to impact host attractiveness, host fitness and disease transmission. Our findings join a small yet growing body of evidence that chytrid fungus impacts frog calling behaviour and more broadly, that pathogens modify host reproductive behaviour.

## Graphical abstract

The sublethal effects of infection and disease on male advertisement behaviour remain poorly understood. Here we show that male brown toadlets infected with the amphibian chytrid fungus produce calls with more pulses compared to uninfected males. The association we report supports the terminal investment hypothesis and has the potential to impact host attractiveness, host fitness and disease transmission.

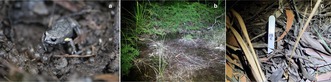

## 1 Introduction

Disease caused by pathogenic infection is known to have lethal consequences for a large diversity of host organisms (Mcknight et al. 2017, O'sullivan et al. 2025). By comparison, the sublethal impacts of infection remain poorly understood. One potentially pervasive sublethal effect relates to individual strategies over resource allocation. Based on the well‐founded assumption that resources are finite, life‐history theory predicts that organisms will face fundamental trade‐offs between investment in life‐history traits that strongly impact fitness (Rauw 2012). A particularly salient trade‐off is the way that resources are invested in resistance to infection relative to investment in reproduction. A conventional notion is that preferential allocation of resources to improve the probability of survival (and future reproduction) will divert resources away from current reproduction (Ashley et al. 2011). Indeed, such trade‐offs have been reported for a diversity of groups, with compelling evidence that infected individuals reduce investment in gametogenesis (Dvorakova‐Hortova et al. 2014; Ford and Figueras 1988; Mccallum and Trauth 2007), sexual advertisement (Comas et al. 2024; Jacot et al. 2004; Kilpimaa et al. 2004) and parental care (Bonneaud et al. 2003; Martínez‐Flores et al. 2024; Råberg et al. 2000). However, an alternative allocation strategy gaining attention is that terminally ill individuals might elevate their lifetime fitness by preferentially investing in current reproduction (Brannelly et al. 2016; Davis et al. 2021; Giehr et al. 2017; Schwanz 2008; Velando et al. 2006). This hypothesis, termed the ‘terminal investment hypothesis’, centres on the idea that infected individuals stand to elevate their lifetime fitness by increasing investment in reproduction when their chance of future reproduction (residual reproductive value) declines (Clutton‐Brock 1984; Duffield et al. 2017; Foo et al. 2023; Parker et al. 2011).

When investigating the ‘terminal investment hypothesis’, it can be expected that there will be sex‐specific differences in investment strategies (Andrade and Kasumovic 2005; Duffield et al. 2017). For males, which typically have a higher Potential Reproductive Rate (PRR) than females and experience stronger selection to attract mates, we might expect infected individuals with reduced residual reproductive value to invest heavily in sexual advertisement. Indeed, there is mounting evidence for such responses across different species and signalling modes (Duffield et al. 2017). For example, male mealworm beetles increase their production of sex pheromones if they are repeatedly immunocompromised (Kivleniece et al. 2010; Krams et al. 2011), and old male decorated crickets significantly increase calling activity when exposed to an infection cue (Duffield et al. 2018). With the goal of better understanding the generality of such responses, there is a need for more studies that investigate the relationship between infection status and male investment in sexual advertisement. Moreover, because reproductive traits can vary in how they respond to cues for terminal investment (e.g., Brannelly et al. (2025), Duffield et al. (2017) and Rutkowski et al. (2023)), it will be informative to study a broad range of sexual signals and signal components. For instance, studies investigating the influence of infection on acoustic advertisement would benefit from exploring investment in calling rate, as well as temporal and spectral qualities of calls that might also influence attractiveness (Gerhardt 1991; Gerhardt and Huber 2002). Ultimately, a more comprehensive approach will facilitate more effective comparisons of male‐advertisement responses across species and, in turn, allow deeper insights into how infection influences mating outcomes, the strengths and targets of sexual selection and population dynamics. Such insights are likely to be especially valuable in taxa challenged by emergent and highly infectious pathogens and diseases that present major threats to biodiversity.

The amphibian chytrid fungus (*Batrachochytrium dendrobatidis*) has spread globally over the past four decades and is responsible for one of the worst wildlife diseases in modern history, driving the extinction of at least 90 amphibian species and the rapid decline of at least 500 others (Scheele et al. 2019). While mortality resulting from pathogen‐induced disease (*Chytridiomycosis*) is a common outcome (Lips 2016; Lips et al. 2006; Rosenblum et al. 2010; Voyles et al. 2011, 2009), for most species there is a period after infection onset where individuals continue to engage in reproductive behaviour (Brannelly et al. 2025, 2016; Kelleher et al. 2021; Messersmith et al. 2024). During this period, there is an opportunity for individuals to strategically modify the way they invest resources in infection defence and somatic maintenance relative to reproduction. Among anuran amphibians (frogs and toads), male reproductive success is generally linked to investment in acoustic signalling, with females typically showing a preference for individuals displaying complex or highly energetic calling behaviour (Forsman and Hagman 2006; Ryan 1988). With an increasing interest in the sublethal impacts of *Bd* infection, the influence of infection on anuran advertisement strategies has recently come into focus. Relationships between *Bd* infection status and calling investment have been investigated in 14 anuran species, and the reported effects are highly varied. Compelling evidence for elevated acoustic advertisement has been reported for six anuran species, including the Australian common mist frog ( *Litoria rheocola* ) and the Australian northern corroboree frog ( *Pseudophryne pengilleyi* ) (An and Waldman 2016; Kelleher et al. 2021; Messersmith et al. 2024; Roznik et al. 2015; Zornosa‐Torres 2021). By contrast, no detectable relationship between *Bd* infection and calling investment has been observed in four species (Brannelly et al. 2025; Zornosa‐Torres 2021), and reduced calling investment has been observed in four species (Rodríguez Brenes 2016; Zornosa‐Torres 2021). Although anurans may exhibit varied responses due to differences in life‐history traits, these apparent differences could also stem from the limited and inconsistent range of call traits measured across these studies. This inconsistency restricts our ability to make effective comparisons across species and draw meaningful conclusions about investment responses. Accordingly, there is a need for more studies that investigate the effects of *Bd* infection on acoustic advertisement in anuran species, with an emphasis on measuring a broad range of call parameters to facilitate more detailed and comprehensive interspecific comparisons.

The aim of the present study was to investigate how *Bd* infection status relates to male calling investment in wild populations of Australian brown toadlets ( *Pseudophryne bibronii* ). This species is displaying population declines across its range, with patterns of local extinction mirroring those reported for chytrid‐susceptible Australian frog species. Specifically, the strongest declines across southeastern Australia have been observed in populations from cool, moist environments where the chytrid fungus is most prevalent (Gillespie et al. 2020; Scheele et al. 2019). With the goal of undertaking a preliminary investigation into the potential for *Bd* to influence calling behaviour, we examined the relationships between infection status and variation in a range of acoustic properties known to influence mating success. In line with the terminal investment hypothesis, we predicted that infected males would display elevated calling investment.

## 2 Materials and Methods

### 2.1 Ethics Statement

All procedures were conducted following approval by the University of Wollongong Ethics Committee, protocol numbers AEPR214 (2023 collections) and AEPR2318 (2024 collections). The scientific research was authorised by the New South Wales National Parks and Wildlife Service and the Department of Planning, Industry and Environment, licence numbers SL102640 and SL102853.

### 2.2 Study Species

**FIGURE 1:**
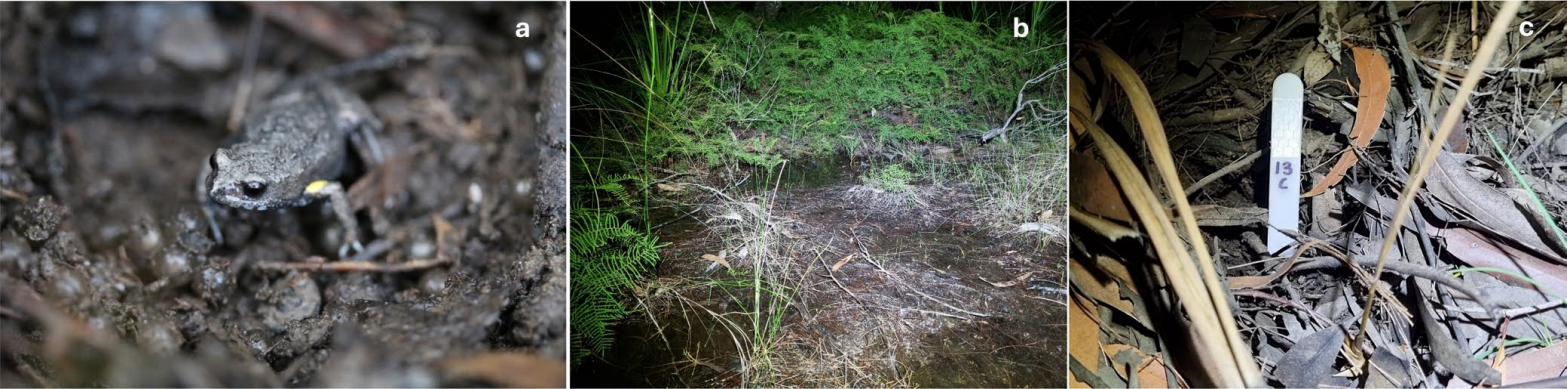
(A) Male brown toadlet, *Pseudophryne bibronii* , in a nest site with eggs. (B) Example of terrestrial breeding habitat for the brown toadlet. (C) Reflective plastic ID tag used to individually mark nest sites. Photographs courtesy of AS.

The brown toadlet ( *Pseudophryne bibronii* ) is a myobatrachid frog (Figure 1a) endemic to temperate regions of south‐eastern Australia (Knight and Tyler 2020). Breeding occurs over autumn to early winter and is triggered by the onset of the first significant autumn rains (Woodruff 1976, 1977). Brown toadlets are terrestrial breeders, with males establishing nesting sites in shallow burrows underneath leaf litter in vegetation adjacent to ephemeral pools and creek lines (Figure 1b) (Woodruff 1977). Within these burrows, males utilise acoustic signalling to advertise their presence to females (Byrne 2008; Pengilley 1971). After rainfall events, females will enter breeding sites and will assess the quality of multiple males over several days before mating (Byrne and Keogh 2009). Females oviposit directly into male nest sites, with males fertilising eggs externally as they enter the nest (Byrne and Keogh 2009). Female brown toadlets are extremely polyandrous and will sequentially split an average total of 47 eggs between nests of up to eight males (Byrne and Keogh 2009; Byrne and Roberts 2012). The embryo develops within egg capsules until Gosner stage 26–27, at which point development is suspended until nest sites are flooded by heavy winter rainfall, and hypoxic conditions trigger hatching (Bradford and Seymour 1988). Larval development occurs over winter in ephemeral water bodies, with metamorphosis occurring in early to mid‐spring (Byrne and Silla 2023; Woodruff 1977). Males will typically continue acoustic advertisement at nest sites over the course of the breeding season until they are inundated by winter rainfall, at which point breeding is finished and males return to surrounding bushland (Byrne and Silla 2023; Heap et al. 2015).

### 2.3 Acoustic Advertisement, Phenotypic Traits and Nest Site Characteristics

**FIGURE 2:**
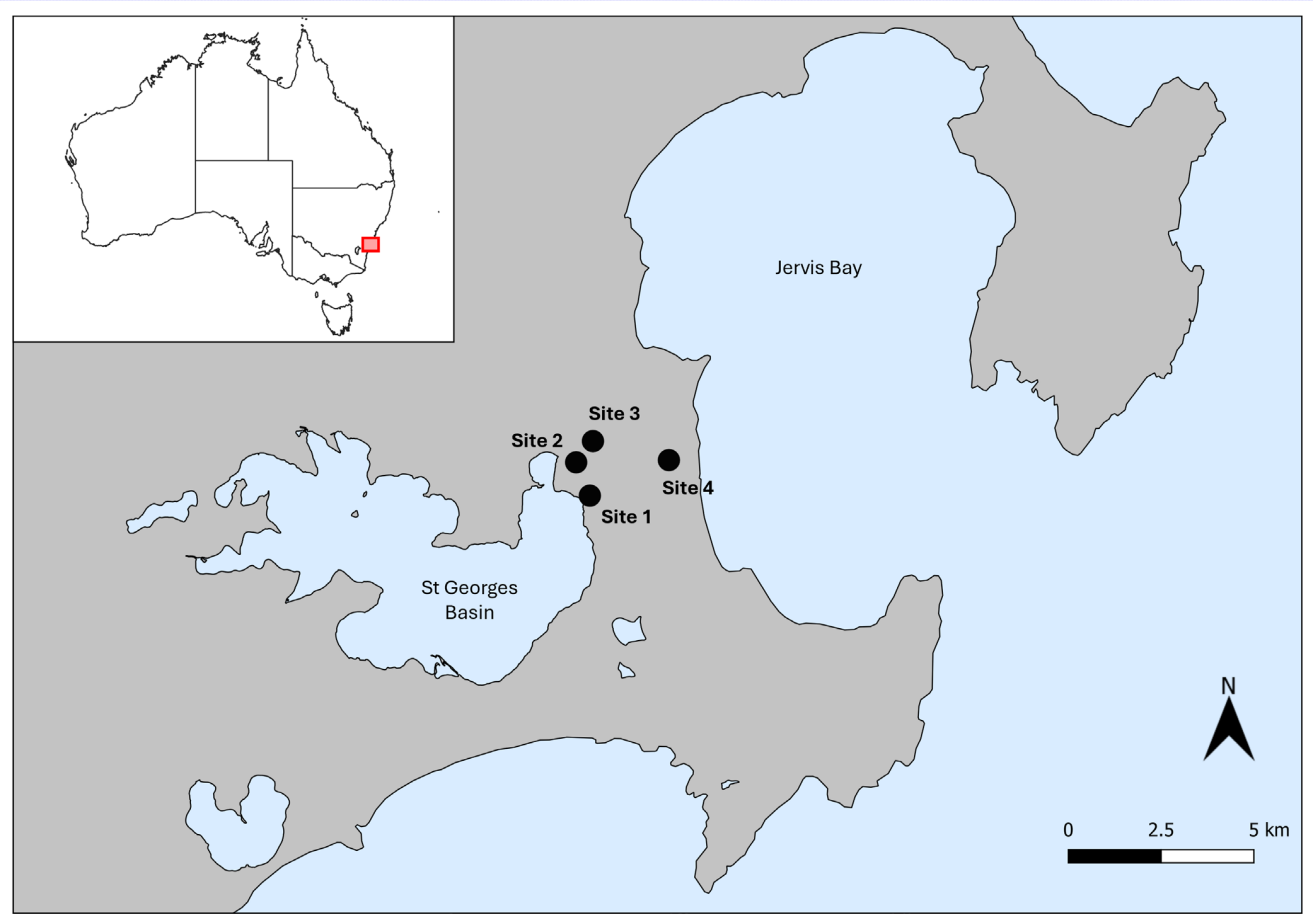
Map showing the locations of the four study sites/populations of brown toadlets, *Pseudophryne bibronii*, located in Jervis Bay National Park, New South Wales, Australia.

This study was conducted during the peak of the species' natural breeding season over two consecutive years (2023 and 2024). Four study sites/populations of brown toadlets were selected within Jervis Bay National Park, NSW, Australia (Figure 2). Sites 1, 2 and 3 were sampled from the 13th to the 16th of April 2023, and Site 4 was sampled from the 16th to the 18th of April 2024. Overall, a total of 60 males were recorded and collected (*n* = 48 in 2023 and *n* = 12 in 2024). During each recording night, male brown toadlets were located through coordinated triangulation of their calls, and nest sites were individually marked with numbered reflective plastic ID tags (Figure 1c). Male vocalisations were then recorded on a Marantz Professional handheld solid‐state recorder (model no. PMD661MKII, D&M Holdings Inc., Tokyo, Japan) using a Rode directional microphone (model NTG2, Sydney, Australia). The directional microphone was positioned approximately 30 cm away from a male's nest site, and recordings were taken in a Pulse‐code Modulation Format (stored as a Waveform Audio File, .wav) at a 44.1 kHz sampling rate with a 16‐bit signal resolution. Call sampling occurred between 18:30 and 23:30, with no sampling occurring after 23:30 to ensure temperatures did not fall below optimal conditions for calling (estimated as 11°C–20°C) (Byrne 2008). Each male was recorded for a minimum duration of 10 advertisement calls. At the beginning of each recording, the ambient air temperature was recorded using a handheld digital thermometer (model SKUHA8963, Marina). Across sampling nights, ambient temperature ranged from 13.3°C to 20°C (mean ± SEM of 16.58°C ± 0.24°C).

Following the collection of audio recordings, males were carefully exhumed from their nest site, placed in individual ventilated plastic containers (9 cm diameter × 5 cm height) and transported to a field station located within 3 km from the furthest collection site. To mitigate the potential for any pathogenic transmission between males, gloves were worn at all times while searching nest sites and handling frogs, with gloves changed between individuals. Additionally, to further prevent the risk of disease transmission between sites, males were only collected and housed from one study site on any given night, with all equipment cleaned and disinfected between sites. Once at the field station, males were swabbed to determine *Bd* infection status (see Section 2.4) and weighed using a digital balance HL‐200i digital compact scale, with male mass ranging from 0.8 to 1.6 g (mean ± SEM of 1.18 ± 0.027). The ventral surface of each frog was photographed next to a ruler using a Panasonic DC‐TZ90 camera (f/3.3, 1/60 s, ISO 125‐500), positioned approximately 20 cm above the frog. Snout‐vent length (SVL) for each frog was later measured in ImageJ software (Rasband, W.S., US National Institute of Health, Bethesda, Maryland, USA) using a linear measurement of SVL length in pixel units which was converted to mm using the ruler scale present in each image. Male SVL ranged from 22 mm to 31 mm (mean ± SEM of 26.22 mm ± 0.28). A body condition index was also later estimated for each male using the residuals of an ordinary least squares regression of body mass on SVL (Schulte‐Hostedde et al. 2005). Across all males, body condition ranged from −0.31 to 0.34 (mean ± SEM of 0.00 ± 0.018). Brown toadlet males were housed in the field station for a maximum of 24 h. During this period, soil moisture was recorded at each nest site using a soil moisture probe and meter (MPM‐160‐B, ICT International Pty Ltd), as nest water content is a known positive correlate of calling rate in brown toadlets (Mitchell 2001). Nest site soil moisture content was moderately to highly saturated across study sites, ranging from 36.5% to 91.7% (mean ± SEM of 57.45% ± 1.73%). Following the completion of phenotypic and nest site measurements, males were then returned to their individual nest sites, with all releases taking place after sunset to reduce the risk of predation.

### 2.4 *Bd* Infection Status

To determine *Bd* infection status, skin swabs were taken from each frog using sterile cotton‐tipped cutaneous swabs (MWE Medical Wire MW100, UK). A standardised swabbing protocol was employed which involved five strokes from each of the following skin surfaces: dorsal surface, ventral surface, left lateral flank, right lateral flank, left armpit to fingers, right armpit to fingers, left inner thigh to toes, right inner thigh to toes (40 strokes total). The swab tip was rotated during collection to ensure coverage of the swab tip. Swabs were labelled and temporarily refrigerated until analysis. Swab samples were analysed for *Bd* infection status and *Bd* zoospore count by Cesar Australia (Cesar Pty Ltd., Victoria, Australia) using a real‐time quantitative polymerase chain reaction (qPCR) with a Qiagen master mix (3 qPCRs per male, total *n* = 180). Samples were considered *Bd‐*positive if one or more qPCRs returned a positive reaction. In the following analyses, *Bd* infection status was categorically defined as either positive or negative for each male.

### 2.5 Male Call Analysis

**FIGURE 3:**
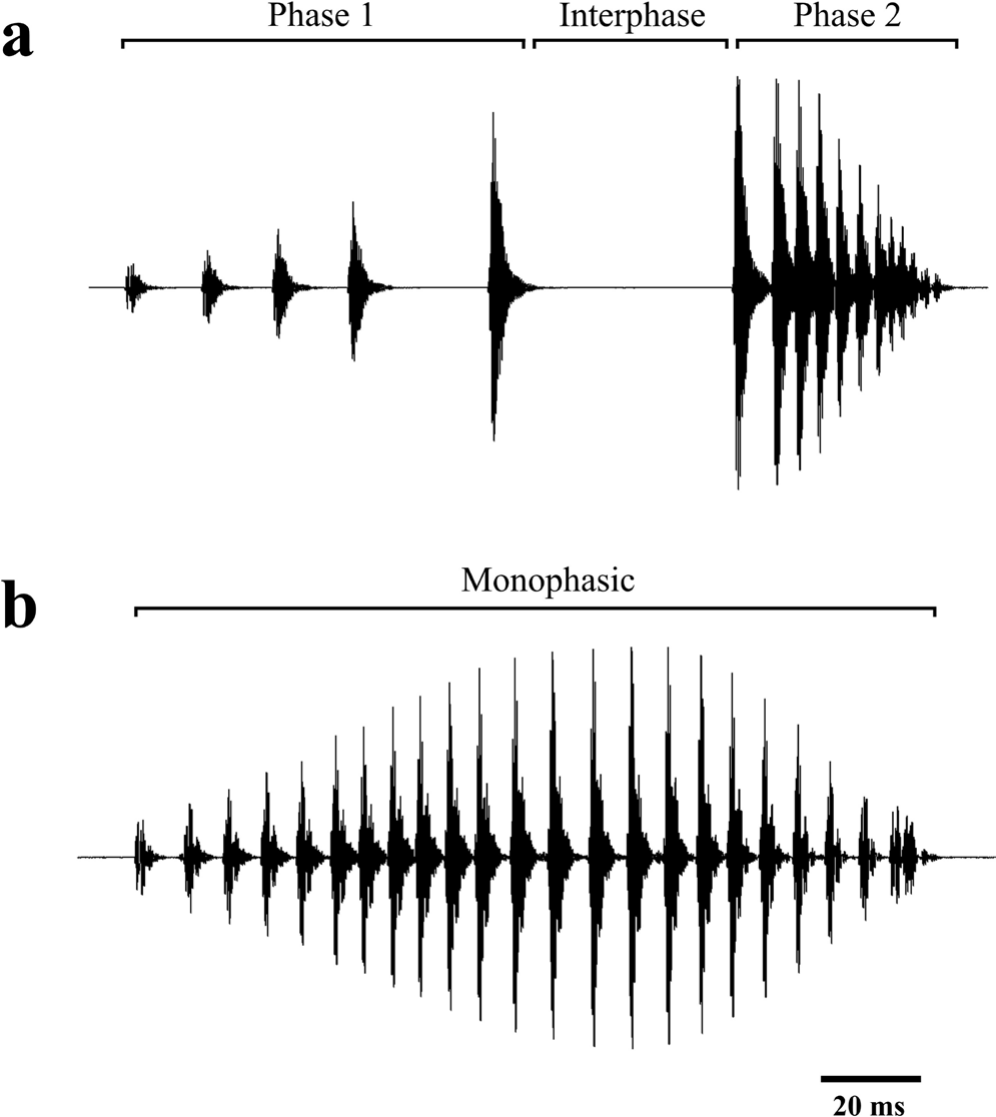
Oscillograms depicting (a) a diphasic advertisement‐type call and (b) a monophasic territorial‐type call produced by a male brown toadlet, *Pseudophryne bibronii* .

Brown toadlet vocalisations can be categorised into advertisement and territorial call types (Byrne 2008). Advertisement calls are biphasic, with the first phase containing typically 1–6 introductory notes, followed by a brief interlude before the second phase of typically 7–15 compact pulses, which descend in amplitude (Figure 3A). Territorial calls are monophasic and consist of one long pulse train where the overall amplitude follows a bell‐curve structure (Figure 3B). Only advertisement calls were analysed in this study as they are the primary proxy for sexual advertisement in brown toadlets. Male advertisement calls were analysed with the bioacoustics software Raven Pro (Version 1.5, Cornell Lab of Ornithology, Bioacoustics Research Program, 2014). Waveform oscillograms were produced in Raven Pro to measure spectral and temporal call parameters. A spectrogram was also produced using a fast Fourier transformation (window = Blackman, window size = 1024, 3‐dB bandwidth = 70.7 Hz, DFT = 2048 samples, grid spacing = 21.5 Hz, with 50% overlap and a hop size of 512 samples) (Kelleher et al. 2021) to undertake measurements of call frequency.

For each of the 60 recorded males, a total of 14 different call parameters were measured: (1) call duration, (2) Phase 1 duration, (3) interphase duration, (4) Phase 2 duration, (5) Phase 1 peak frequency (frequency of the dominant amplitude of the call, measured in Hertz, Hz), (6) Phase 2 peak frequency, (7) total pulse number, (8) Phase 1 pulse number, (9) Phase 2 pulse number, (10) call pulse repetition rate (calculated as the number of pulses per second), (11) Phase 1 pulse repetition rate, (12) Phase 2 pulse repetition rate, (13) call rate (calculated as the number of calls produced per minute) and (14) call effort (a metric in pulses per minute which was calculated as the average total pulse number multiplied by the call rate). Following Koehler et al. (2017), call pulses were defined as single sound energy bursts separated by strong amplitude modulation from other pulses. As such, low‐amplitude pulsatile structures with minimal modulation, typically located towards the trailing end of advertisement calls, were not categorised as pulses. All call analyses were performed by one researcher. Summary statistics of the 14 sampled call parameters can be found in Table A1.

### 2.6 Statistical Analysis

All statistical analyses were performed using R statistical software (R Core Team 2025). A total of six explanatory variables were selected for the analysis. These explanatory variables were *Bd* infection status (positive or negative), ambient temperature (°C), soil moisture (%), SVL (mm), mass (g) and site. Before conducting statistical analyses, a Pearson correlation matrix was used to test for any collinearity between the continuous explanatory variables. Any pairs of variables correlated by a value of *R* > 0.7 were subjected to selective exclusion (Dormann et al. 2013). Mass and SVL were found to be significantly correlated (*R* = 0.74), so mass was excluded from the analysis. Snout–vent length is preferred over mass as a measure of anuran body size because it is more consistent across different body conditions and provides a more stable indicator of core anatomical size (Santini et al. 2018). In addition, we checked for associations between ambient temperature (°C), soil moisture (%), SVL (mm), body condition and *Bd* infection status using logistic regressions. None of these variables predicted *Bd* infection status (Table A2), so no interaction terms were included in subsequent analyses.

Prior to statistical analysis, the mean for each call trait (*n* = 14 traits) was obtained from 10 sequential advertisement calls for each male. To account for correlations between the 14 call traits and reduce the dimensionality of the data set, we performed a Principal Components Analysis (PCA). To investigate the potential relationship between *Bd* infection status and the male call traits, linear mixed effects (LME) models were conducted in R statistical software using the ‘lme4’ package (Bates et al. 2015). The fixed effects were ambient temperature, SVL, body condition, *Bd* infection status and soil moisture. Site was specified as a random effect. The response variables were the principal component scores for three PCs from the PCA analysis. The principal components were selected based on those that had eigenvalues greater than one (following the Kaiser criterion) and by observing a scree plot which showed a clear point of diminishing returns (elbow) beyond three principal components. Normality was assessed using a Shapiro–Wilk test, and homoscedasticity was assessed by plotting residuals. The *p* values of the fixed effects were computed using a Type III analysis of variance table following Satterthwaite's method (Kuznetsova et al. 2017). An alpha level of *p* < 0.05 was selected for determining statistical significance.

## 3 Results

Across the four study sites (and across the two study years), 10% (6/60) of male brown toadlets were found to be infected with *Bd*. In the first study year, *Bd* was detected in three study sites, and in the second study year, *Bd* was detected in one study site (Table A3). The infection load between individuals was highly variable, with the zoospore count ranging between 7 and 536,667 zoospores (mean ± SEM = 97,967.83 ± 87,897.79). However, half of the infected males had zoospore counts above the level (10,000 zoospores) expected to lead to disease and mortality (Vredenburg et al. 2010).

A PCA of the 14 call traits resulted in three principal components with Eigen values greater than one, which together explained 68.44% of the variance. Principal Component 1 was dominated by call pulse number, call duration, Phase 1 pulse number, and Phase 1 duration (Eigen value = 4.56, % variance = 32.58); Principal Component 2 was dominated by call pulse repetition rate, Phase 2 pulse number and Phase 2 duration (Eigen value = 2.90, % variance = 20.68); and Principal Component 3 was dominated by Phase 1 peak frequency and Phase 2 peak frequency (Eigen value = 2.13, % variance = 15.19).

*Bd* infection status was found to have a significant effect on Principal Component 1 (LME: *F*_1,53.32_ = 5.068, *p* = 0.029). This result reflects a relationship whereby males infected with *Bd* produced calls that contained more pulses than calls produced by uninfected males (Table 1). Principal Component 1 was not significantly affected by ambient temperature (LME: *F*_1,48.13_ = 3.209, *p* = 0.080) or soil moisture (LME: *F*_1,52.52_ = 0.019, *p* = 0.891). However, Principal Component 1 was significantly affected by male SVL (LME: *F*_1,51.84_ = 4.989, *p* = 0.030) and male body condition (LME: *F*_1,53.93_ = 5.768, *p* = 0.020), reflecting a relationship whereby larger males and males in better body condition produced calls with more pulses.

**TABLE 1:** Effect of *Bd* infection status on call traits in wild brown toadlets*, Pseudophryne bibronii*.

Call traits	*Bd* infection status	
	Infected	Not infected
Call duration (sec)	0.151 ± 0.00488	0.149 ± 0.00283
Phase 1 duration (sec)	0.0541 ± 0.00469	0.0522 ± 0.00280
Interphase duration (sec)	0.0368 ± 0.00246	0.0400 ± 0.00127
Phase 2 duration (sec)	0.0601 ± 0.00357	0.0581 ± 0.00113
Phase 1 peak frequency (Hz)	2645.00 ± 73.177	2544.63 ± 24.610
Phase 2 peak frequency (Hz)	2681.61 ± 48.176	2625.92 ± 24.521
Call pulse number	14.867 ± 0.541	13.426 ± 0.283
Phase 1 pulse number	2.967 ± 0.219	2.974 ± 0.138
Phase 2 pulse number	11.900 ± 0.501	10.452 ± 0.245
Call pulse repetition rate (pulses/s)	98.562 ± 1.634	90.778 ± 1.610
Phase 1 pulse repetition rate (pulses/s)	58.963 ± 2.331	65.708 ± 2.213
Phase 2 pulse repetition rate (pulses/s)	202.096 ± 9.806	181.157 ± 2.765
Call rate (calls/min)	14.667 ± 3.480	13.704 ± 0.831
Call effort (pulses/min)	218.1 ± 52.19	187.27 ± 13.62

*Note:* Data shown are untransformed mean ± SEM (*n* = 60).

*Bd* infection status had no significant effect on Principal Component 2 (LME: *F*_1,54_ = 3.224, *p* = 0.078). Principal Component 2 was also not significantly affected by male SVL (LME: *F*_1,54_ = 0.756, *p* = 0.388), ambient temperature (LME: *F*_1,54_ = 0.001, *p* = 0.974) or soil moisture (LME: *F*_1,54_ = 0.118, *p* = 0.733). However, Principal Component 2 was significantly affected by body condition (LME: *F*_1,54_ = 4.031, *p* = 0.0497), reflecting a similar relationship to PC1, with males in better body condition producing calls with higher pulse repetition rate and a greater number of pulses in Phase 2.

*Bd* infection status had no significant effect on Principal Component 3 (LME: *F*_1,53.27_ = 0.460, *p* = 0.501). Principal Component 3 was also not significantly affected by male body condition (LME: *F*_1,53.98_ = 0.006, *p* = 0.941), ambient temperature (LME: *F*_1,45.71_ = 0.065, *p* = 0.799) or soil moisture (LME: *F*_1,51.64_ = 3.582, *p* = 0.064). However, Principal Component 3 was significantly related to male SVL (LME: *F*_1,51.40_ = 7.953, *p* = 0.007), reflecting larger males having a lower peak frequency in Call Phases 1 and 2.

## 4 Discussion

Whether males elevate their investment in reproduction in response to pathogenic infection, as predicted by the ‘terminal investment hypothesis’, is increasingly being investigated. Gaining this knowledge is particularly valuable for understanding the impacts of highly virulent emerging infectious diseases. The amphibian chytrid fungus (*Batrachochytrium dendrobatidis*) is now globally widespread and is driving the decline of hundreds of species, but we are only just beginning to explore the impacts of infection on anuran calling behaviour. The aim of the present study was to provide a preliminary investigation into the influence of *Bd* infection on advertisement calling in the Australian brown toadlet, *Pseudophryne bibronii* . After controlling for the influence of extrinsic and intrinsic variables (temperature, soil moisture, body size, body condition) known to influence calling behaviour, our results indicate that infected males have a significantly higher number of pulses per call.

Despite detecting a relatively low infection level across our study populations, the differences in calling investment between infected and uninfected males were significant. Overall, infected males produced advertisement calls that contained approximately 11% more pulses. This result suggests that *Bd* infection may be influencing male‐resource allocation, with a shift towards investment in current reproduction. These findings provide support for the ‘terminal investment hypothesis’, which proposes that males will elevate investment in reproductive traits as their reproductive value declines (Clutton‐Brock 1984; Duffield et al. 2017; Foo et al. 2023; Parker et al. 2011). At present, we have no data on how quickly brown toadlets die after they are infected with *Bd*, so it is unclear how male relative reproductive value is likely to change in the period after infection. Saying this, it is noteworthy that none of the infected males in our study displayed characteristics of moribund frogs (e.g., emaciation, redness, body tremors, lethargy). Therefore, changes in advertisement appear to have occurred before males experienced major drops in reproductive value. It should be recognised, however, that brown toadlets are iteroparous, with breaks of 6 months or more between breeding seasons (Byrne 2008; Byrne and Keogh 2009). Observations of *Bd* impacts in related species (e.g., northern corroboree frog, *Pseudophryne pe* *ngilleyi* , and southern corroboree frog, *Pseudophryne corroboree* ) suggest that infected individuals tend to die within a few months (Brannelly et al. 2015; Davidson et al. 2025). Assuming this is also true for brown toadlets, infected males may be unlikely to survive until the following breeding season, placing a selective premium on a rapid increase in sexual advertisement during the current breeding season. Given that our study took place at the start of the breeding season, it would be insightful to assay the behaviour of males towards the end of the season, when residual reproductive value is likely to have further declined. If males are terminally investing, we should expect even stronger investment in calling by infected males towards the end of the breeding season.

While our findings support the ‘terminal investment hypothesis’, it is important to recognise that our data is correlational, and that there may be alternative explanations for the patterns we observed. One possibility to consider is that increased calling investment is mediated by the pathogen rather than the host. The ‘parasite manipulation hypothesis’ proposes that pathogens can manipulate their host behaviours to maximise the potential for transmission between individuals (Adamo and Hughes 2012; Herrera and Nunn 2019; Klein 2003). Following this model, increased calling behaviour in *Bd*‐infected male brown toadlets could facilitate increased contact between infected males and uninfected females (Hernandez‐Caballero et al. 2022). While this remains a plausible alternative hypothesis, there is currently no direct evidence identifying a neurological mechanism that would facilitate host manipulation by *Bd* in amphibians. Furthermore, there is emerging evidence that male frogs infected with *Bd* invest in increased testes size and sperm performance (Brannelly et al. 2021, 2025, 2016; Chatfield et al. 2013). Because these traits are expected to improve male reproductive success, yet have little or no impact on *Bd*‐transmission rates, it is hard to argue that host manipulation, rather than terminal investment, is the primary mechanism underpinning elevated calling investment. Nonetheless, to exclude this possibility, future studies should utilise nonpathogenic immune stimulation to distinguish between host‐mediated responses and pathogen manipulation (Duffield et al. 2017). Any observed increases in calling investment under nonpathogenically triggered immune responses could be confirmed as host‐mediated rather than pathogen‐mediated (Duffield et al. 2017). While this approach has been applied across various other taxa, such as birds (Bonneaud et al. 2004; Bowers et al. 2015; Sköld‐Chiriac et al. 2019), mammals (Derting and Virk 2005) and insects (Krams et al. 2011), it is yet to be employed in amphibian research.

An alternative explanation for our findings is that the positive associations observed are reversely causal. In essence, males that exhibit naturally high calling investment may be at greater risk of *Bd* infection, rather than increasing investment after infection onset (An and Waldman 2016). This could occur, for instance, if higher quality males that advertise more are predisposed to more social interactions, or are moving more through a breeding site and are more likely to encounter the pathogen. To date, no study in amphibians has clarified whether males that invest more in calling are at higher risk of being infected with chytrid. To this end, it would be valuable for future studies to monitor calling investment before, during, and after *Bd* infection. This would enable us to ascertain whether high calling investment is static over the course of infection or triggered by infection onset. Undertaking such research will be an important step forward as it will allow us to draw stronger conclusions about the mechanism driving elevated calling in *Bd*‐infected males.

Irrespective of the explanation for elevated calling investment, our findings add to a small but growing list of anuran species where males have been reported to modify calling investment in response to *Bd* infection. To date, elevated investment in acoustic advertisement has been documented in six frog species, with impacts on various call traits (and combinations of traits) reported. Infected males have been shown to have a higher call pulse repetition rate in the northern corroboree frog (*Pseudophyrne pengilleyi*) (Kelleher et al. 2021), higher call pulse repetition rate in Pacific tree frogs ( *Pseudacris regilla* ) (Messersmith et al. 2024), higher call pulse repetition rate and call duration in Japanese tree frogs ( *Hyla japonica* ) (An and Waldman 2016) and higher call duration and call rate in both the lesser tree frog ( *Dendropsophus minutus* ) and the Crubixa snouted tree frog ( *Scinax alter* ) (Zornosa‐Torres 2021). Some more nuanced changes linked to male phenotype have also been reported. In the common mist frog ( *Litoria rheocola* ), it has been observed that good‐condition males have a higher probability of calling when infected with *Bd* (Roznik et al. 2015), without showing any difference in temporal or spectral properties of individual calls (Greenspan et al. 2016). Such interspecific variation suggests that anurans may respond to *Bd* infection by modifying a broad diversity of call traits. Intuitively, such variable responses should be expected given that anurans display considerable interspecific variation in reproductive ecology and the specific call traits that most strongly influence male attractiveness and patterns of female‐mate choice (Duellman and Trueb 1994). However, it should be emphasised that the aforementioned studies did not consistently measure the same suite of call traits. Therefore, it remains unclear whether interspecific variation reflects different evolutionary histories and reproductive ecologies, or simply incomplete sampling of the various call traits potentially influenced by *Bd* infection. Moving forward, we strongly suggest that future studies measure a standardised set of call traits known to influence anuran mating outcomes. This approach will facilitate interspecific comparisons that enable more in‐depth investigation into how male frogs modify their calling behaviour in response to *Bd* infection, and whether interspecific differences in reproductive ecology mediate different responses.

Given that elevated calling investment associated with *Bd* infection has been reported in seven frog species (including brown toadlets) spanning three different anuran families (Myobatrachidae, Hylidae and Pelodryadidae), such behavioural responses may be widespread in this vertebrate class. If this proves to be the case, *Bd* could have direct effects on male‐mating success and have major, yet currently unappreciated, ecological and evolutionary consequences for anuran amphibians. At present, only two studies have investigated links between elevated calling in infected males and mating success. In their study of northern corroboree frogs, Kelleher et al. (2021) reported that infected males received more eggs in their nest. By contrast, in their study of Pacific tree frogs, two‐choice tests showed that females displayed no preference for calls resembling males with high and low infection levels (Messersmith et al. 2024). Assuming that females do not discriminate between the calls of infected or uninfected males, or even prefer the calls of infected males, then recruitment may stabilise or increase in *Bd*‐infected populations (Brannelly et al. 2025; Valenzuela‐Sánchez et al. 2022). Indeed, such compensatory recruitment has been proposed to be one of the mechanisms underpinning the persistence of populations that harbour *Bd* (Brannelly et al. 2021; Muths et al. 2011; Palomar et al. 2023; Phillott et al. 2013; Scheele et al. 2015, 2017; Tobler et al. 2012; Valenzuela‐Sánchez et al. 2022; West et al. 2020). Compensatory recruitment may also have long‐term evolutionary consequences (Brannelly et al. 2016; Valenzuela‐Sánchez et al. 2022). For example, if *Bd*‐infected males achieve greater mating success as an outcome of elevated reproductive investment, selection for *Bd* resistance could be relaxed (Brannelly et al. 2016). In theory, this could have catastrophic consequences for small populations. While populations might remain stable while recruitment remains high, a single breeding season with no or limited reproduction and recruitment (e.g., due to low rainfall or extreme temperatures) could extirpate populations (Brannelly et al. 2016). There is also some evidence to suggest that compensatory recruitment from *Bd* infection can lead to population demographic changes. For example, a study in the Australian alpine tree frog ( *Litoria verreauxi alpina* ) found that *Bd*‐infected populations experienced high adult mortality, with individuals only breeding once, as opposed to over multiple years as observed in disease‐free populations (Valenzuela‐Sánchez et al. 2022). Under such conditions, the benefits of terminal investment may strengthen selection for males to breed at a young age. This could make older males less valuable, and select against longevity, driving rapid life‐history evolution in diseased populations (Valenzuela‐Sánchez et al. 2022). Given these possibilities, future research should focus on monitoring changes in recruitment, age demographics and disease resistance in populations harbouring *Bd*. Such information will improve our understanding of the ecological and evolutionary consequences of reproductive change linked to the chytrid fungus pandemic and assist with the management of threatened species.

In conclusion, the sublethal impacts of amphibian chytrid fungus infection and chytridiomycosis disease are only just starting to be explored. In line with the ‘terminal investment hypothesis’, a small number of studies have reported that male anurans elevate investment in advertisement calling in response to infection. Our preliminary study with brown toadlets suggests that this species might also be adopting a terminal investment strategy by increasing investment in calling post infection. As for previous anuran studies, the approach we employed was unable to rule out pathogen‐mediated manipulation of host behaviour or the observed relationship being reversely causal. Therefore, experimental studies that allow us to differentiate between these potential mechanisms will be an important future research direction. Regardless of the cause, if elevated advertisement is a widespread response of male anurans to *Bd* infection, this sublethal effect could have significant ecological and evolutionary implications. We encourage ongoing investigation into male advertisement responses to *Bd* infection across a broader diversity of species to deepen our knowledge of the interplay between *Bd* infection, amphibian reproductive ecology and reproductive‐allocation strategies.

## Author Contributions

**Ewan S. Auld:** data curation (equal), formal analysis (lead), investigation (equal), methodology (supporting), validation (equal), visualization (equal), writing – original draft (lead), writing – review and editing (equal). **Aimee J. Silla:** conceptualization (equal), data curation (equal), funding acquisition (lead), investigation (equal), methodology (equal), project administration (equal), resources (equal), supervision (equal), validation (equal), visualization (equal), writing – review and editing (equal). **Phillip G. Byrne:** conceptualization (equal), data curation (equal), formal analysis (supporting), investigation (equal), methodology (equal), project administration (equal), resources (equal), supervision (equal), validation (equal), visualization (equal), writing – original draft (supporting), writing – review and editing (equal).

## Conflicts of Interest

The authors declare no conflicts of interest.

## Supporting information


**Data S1:** ece372354‐sup‐0001‐DataS1.csv.

**Data S2:** ece372354‐sup‐0002‐DataS2.txt.

**Data S3:** ece372354‐sup‐0003‐DataS3.R.

## Data Availability

The original data contributions presented in the study are included in the article and Supporting Information. Further inquiries can be directed to the corresponding author.

## Appendix A

**TABLE A1:** Summary statistics of the 14 sampled call parameters from 60 male brown toadlets, *Pseudophryne bibronii* .

Call Parameter	Mean ± SEM	Minimum	Maximum
Call duration (sec)	0.149 ± 0.00259	0.1085	0.1987
Phase 1 duration (sec)	0.0524 ± 0.00255	0.0124	0.1125
Interphase duration (sec)	0.0397 ± 0.00117	0.0196	0.0615
Phase 2 duration (sec)	0.0583 ± 0.00107	0.0390	0.0782
Phase 1 peak frequency (Hz)	2554.67 ± 23.46	1972.44	3006.04
Phase 2 peak frequency (Hz)	2631.49 ± 22.59	2228.69	3068.46
Call pulse number	13.57 ± 0.26	8.8	17.2
Phase 1 pulse number	2.97 ± 0.13	1.2	5.5
Phase 2 pulse number	10.60 ± 0.23	6.9	15.1
Call pulse repetition rate (pulses/s)	91.56 ± 1.49	69.97	118.27
Phase 1 pulse repetition rate (pulses/s)	65.03 ± 2.02	42.01	130.65
Phase 2 pulse repetition rate (pulses/s)	183.25 ± 2.77	143.85	242.03
Call rate (calls/min)	13.8 ± 0.81	5	36
Call effort (pulses/min)	190.4 ± 13.21	63.5	558

**TABLE A2:** Output of individual logistic regressions of the explanatory variables against *Bd* infection status.

Variable	*χ* ^2^_df_	*p*
Ambient temperature	1.780_1_	0.182
Soil moisture	3.319_1_	0.068
SVL	2.976_1_	0.085
Body condition	2.220_1_	0.136

**TABLE A3:** Effect of *Bd* infection status on call traits in wild brown toadlets*, Pseudophryne bibronii*.

Site	Year	*Bd* Infection Status	
		Number of infected males	Number of uninfected males
Site 1	2023	1	24
Site 2	2023	1	7
Site 3	2023	3	12
Site 4	2024	1	11
